# Hybrid Polydimethylsiloxane (PDMS) Incorporated Thermogelling System for Effective Liver Cancer Treatment

**DOI:** 10.3390/pharmaceutics14122623

**Published:** 2022-11-28

**Authors:** Panqin Ma, Lu Jiang, Xi Luo, Jiayun Chen, Qi Wang, Ying Chen, Enyi Ye, Xian Jun Loh, Caisheng Wu, Yun-Long Wu, Zibiao Li

**Affiliations:** 1 Fujian Provincial Key Laboratory of Innovative Drug Target Research and State Key Laboratory of Cellular Stress Biology, School of Pharmaceutical Sciences, Xiamen University, Xiamen 361102, China; 2 Department of Biomedical Engineering, School of Biomedical and Pharmaceutical Sciences, Guangdong University of Technology, Guangzhou 510006, China; 3 BE/Phase I Clinical Center, The First Affiliated Hospital of Xiamen University, School of Medicine, Xiamen University, Xiamen 361000, China; 4 Institute of Sustainability for Chemicals, Energy and Environment (ISCE2), A*STAR (Agency for Science, Technology and Research), 1 Pesek Road, Jurong Island, Singapore 627833, Singapore; 5 Institute of Materials Research and Engineering, A*STAR (Agency for Science, Technology and Research), Singapore 138634, Singapore

**Keywords:** thermal response, sustained drug release, in situ, injectable hydrogel

## Abstract

For the delivery of anticancer drugs, an injectable in situ hydrogel with thermal responsiveness and prolonged drug release capabilities shows considerable potential. Here, we present a series of thermosensitive in situ hydrogels that serve as drug delivery systems for the treatment of liver cancer. These hydrogels were created by utilizing the polydimethylsiloxane (PDMS) oligomer, polyethylene glycol (PEG) and polypropylene glycol (PPG)’s chemical cross-linking capabilities. Doxorubicin (DOX) was encapsulated in a hydrogel with a hydrophobic core and hydrophilic shell to enhance DOX solubility. Studies into the behavior of in situ produced hydrogels at the microscopic and macroscopic levels revealed that the copolymer solution exhibits a progressive shift from sol to gel as the temperature rises. The hydrogels’ chemical composition, thermal properties, rheological characteristics, gelation period, and DOX release behavior were all reported. Subcutaneous injection in mice was used to confirm the injectability. Through the in vitro release of DOX in a PBS solution that mimics the tumor microenvironment, the hydrogel’s sustained drug release behavior was confirmed. Additionally, using human hepatocellular hepatoma, the anticancer efficacy of thermogel (DEP-2@DOX) was assessed (HepG2). The carrier polymer material DEP-2 was tested for cytotoxicity using HepG2 cells and its excellent cytocompatibility was confirmed. In conclusion, these thermally responsive injectable hydrogels are prominent potential candidates as drug delivery vehicles for the treatment of hepatocellular carcinoma.

## 1. Introduction

Liver cancer is a global health problem with increasing incidence and mortality, making it one of the top ranked causes of death related to cancer [1]. It has been assessed that there were 900,000 new cases and 830,000 deaths from liver cancer in 2020, and it is estimated that more than 1 million individuals will be affected by liver cancer annually by 2025 [2]. There have been multiple developments in the treatment of liver cancer, which include chemotherapy [3], surgical interventions, gene-based therapy [4], and immunotherapy [5], or a combination of them. It is estimated that chemotherapy remains the primary treatment option for approximately 50–60% of patients with liver cancer, although the implementation of surgical and local treatments continues to expand globally. Nevertheless, the majority of chemotherapy drugs present a risk of immediate or chronic toxic effects [6]. Taking the anthracycline antibiotic doxorubicin (DOX) as an instance, it is an antineoplastic agent with a wide spectrum and has been used for many years in the treatment of liver cancer with good results [7]. Unfortunately, the incidence of cardiotoxicity and drug resistance of DOX is remarkably significant [8], subsequently restricting the clinical application [9,10].

In an effort to minimize the risk of toxic side effects and improve the efficacy of DOX, there have been various drug delivery systems (DDS) developed, including microspheres [11], liposomes, polymeric micelles [12], and hydrogels [13,14]. One of the prominent controlled release drug systems is based on polymers owing to the unique pharmacokinetic properties of hydrogels [15]. It is particularly important that in situ forming hydrogel systems are becoming more and more popular as candidates for injectable agents and cellular delivery vectors [16]. Compared with traditional injectable hydrogels, stimulus-responsive hydrogels have attracted considerable attention, with properties such as being pH-responsive [17], temperature-sensitive [18,19,20], magnetic-responsive [21], acoustically-responsive [22], and optically-responsive [23,24]. In turn, considerable progress has been made in development of these fields [25]. Particularly, it is reported that temperature-sensitive responsive hydrogels are more favorable for the formation of hydrogels at the pathological areas, leading to the expectation of effective drug release [26,27]. However, it is still an ongoing challenge to design a slow-release temperature-responsive hydrogel for drug delivery in liver cancer therapy due to the complexity of the tumor microenvironment.

**Scheme 1:**
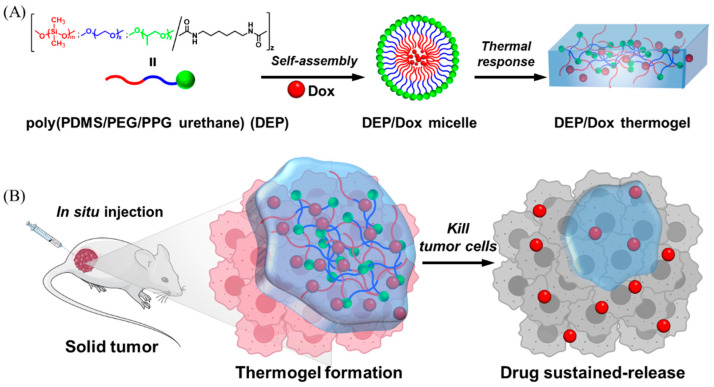
Improves the antitumor effect of DOX by forming hydrogels in situ at the tumor site through thermo responsive behavior of PDMS based polyurethanes for sustained drug release. (**A**) The molecular structure of copolymer DEP and the preparation of its thermal gel. (**B**) The slow release of drug from the thermal gel after in situ injection to achieve effective anti-tumor action.

A series of temperature-responsive injectable hydrogels were developed on the basis of polydimethylsiloxane-*b*-poly (ethylene glycol)-*b*-poly (propylene glycol) copolymers (poly(PDMS/PEG/PPG polyurethanes)) within the context of the present study (Scheme 1). As a soft segment of a polyurethane block polymer, PDMS has excellent oxidation resistance, thermal stability, favorable biocompatibility and low surface tension [28]. Temperature-sensitive hydrogels could be formed by PDMS and temperature-sensitive PEG and PPG fragments, which have attracted attention and are widely used in the design of drug delivery carriers and tumor therapy [29]. The copolymers were optimized to encapsulate the chemotherapeutic drug DOX with controlled release behavior. It had been preliminarily demonstrated to have potential to be used as a delivery carrier for DOX in the treatment on liver cancer. We prepared poly(PDMS/PEG/PPG urethane) copolymer solution in physiological saline at room temperature. The chemical structure, internal morphology, rheological properties, critical micelle concentration (CMC), thermo-dependent degradation and kinetics of drug release, time to gelation and injection properties were assessed.

## 2. Materials and Methods

### 2.1. Materials

Poly(dimethylsiloxane) bis(hydroxyalkyl) terminated (PDMS-diol, M_n_ = 5600 g mol^−1^), poly(ethylene glycol) (PEG, M_n_ = 2050 g mol^−1^), poly(propylene glycol) (PPG, M_n_ = 2000 g mol^−1^), dibutyltin dilaurate (DBT, 95%), 1,6-hexamethylene diisocyanate (HMDI, 98%) and toluene (anhydrous, 99.8%) were purchased from Sigma Aldrich (Merck, Germany). Doxorubicin hydrochloride (DOX, 98%) and 3-(4,5-dimethylthiazol-2-yl)-2,5-diphenyl tetrazolium bromide (MTT, 98%) was obtained from Yuanye Bio-Technology (Shanghai, China). TUNEL staining and Annexin-PI cell apoptosis kit were obtained from Yeasen (Shanghai, China). In addition, all other chemicals are of either analytical or chromatographic grade and require no further purification.

The proton (^1^H) Nuclear Magnetic Resonance (NMR) spectra were recorded in a room environment with a JEOL Ultrashield 500 MHz NMR spectrometer. With CDCl_3_ serving as solvent, an internal reference of tetramethylsilane (TMS) was used. The prepared polymers’ molecular weights and polydispersity exponents were measured by gel permeation chromatography (GPC). GPC was conducted by employing a Viscoteck GPC max module fitted with a refractive index detector and dual Phenogel columns (10^3^ and 10^5^ Å) (size: 300 × 7.80 mm^2^). Polymethyl methacrylate (PMMA) was used as the standard using tetrahydrofuran (THF) as one of the eluents with a flow velocity of 1.0 mL/min at 40 °C. Fourier transform infrared (FT-IR) measurement of copolymer was performed on a Spectrum 2000 Perkin Elmer FT-IR spectrophotometer (Perkin Elmer, Boston, MA, USA) in room conditions. FTIR spectra were recorded in the range of wavenumbers from 4000–400 cm^−1^ with 32 signal averaging scans and a resolution of 4 cm^−1^. The thermogravimetric analysis (TGA) of poly(PDMS/PEG/PPG urethane) was conducted using the TA Instruments TGA Q500 analyzer (USA). The decomposition temperatures (T_d_) were recorded in a N_2_ atmosphere with a flow rate of 50 mL min^−1^ and a temperature of 800 °C for the copolymers. A Q100 photo differential scanning calorimeter was used (PDSC, TA Instruments, New Castle, DE, USA). As the calibration, indium was utilized. It is balanced at −80 °C, then isothermal in −80 °C with 5 min, ramped up from −80 °C to +200 °C at 20 °C/min (first heating cycle), equilibrated at +200 °C for 2 min, and then ramped up from +200 °C to −80 °C with 20 °C/min (first cooling cycle), isothermal at −80 °C for 5 min, eventually rising again to +200 °C by 20 °C/min (second heating cycle). The data chosen for computation and analysis were from the first cooling cycle and second heating cycle. Additionally, a high-sensitivity laser confocal microscope (LSM5, Zeiss, Germany) was used.

### 2.2. Methods

#### 2.2.1. Copolymer Synthesis of Poly(PDMS/PEG/PPG Urethane)

Poly(PDMS/PEG/PPG urethane) copolymers with three different weight percentages of PDMS-diol component (2, 5 and 8 wt%) and fixed PEG and PPG oligomer ratio (2:1) have been synthesized following our previous procedure [14,30]. The synthesized poly(PDMS/PEG/PPG urethane) copolymers are named DEP-*n*, and D stands for PDMS-diol, E stands for PEG and P stands for PPG. The fixed oligomers of PEG and PPG (10 g, feed ration = 2:1), and different amounts of PDMS-diol were first weighed into the same flask, then the polymer was pre-dried by placing the flask into a reduced vacuum at 40 °C overnight. The system was added to anhydrous toluene (100 mL) and then the mixture was further dried twice on a rotary evaporator (Buchi R-210, Flawil, Switzerland) by azeotropic distillation up to a remaining small amount of toluene. Under argon atmosphere, HMDI (0.84 mL) and a catalytic amount of DBT were injected into the system after heating the reactive mixture to 110 °C. After 24 h, the response mixture was quenched by methanol (5 mL) and cooled down, then precipitated two times into ether/hexane mixed solvent (3:7 *v*/*v*) to provide the crude product poly(PDMS/PEG/PPG urethane). The crude product was dissolved in isopropanol (IPA) and purified with dialysis in distilled deionized water (DI) over a period of 3 days; the aqueous solution was then freeze-dried to provide a fluffy solid polyurethane with a yield of 70–90%. The detailed feed ratios and components, and molecular characterization data including ^1^H NMR and GPC of each synthesized polyurethane have been calculated and are summarized in Table 1.

#### 2.2.2. Transitional Characteristics of Sol-Gel

The sol-gel transition behaviors of the generated copolymers were assessed with heat ranges from 4 to 80 °C, using the previously described inverted vial technique [31]. The glass vials were first filled with an aqueous copolymer solution in a range of concentrations between 2 and 20 wt%. The solutions were then allowed to equilibrate at 4 °C for 24 h. The completely dissolved copolymer aqueous solution specimens were immersed in a liquid bath and gradually heated up to 80 °C from 4 °C at intervals of 2 °C with an equilibration time of 5 min at each temperature point. When a hard gel had formed and was able to remain intact after being inverted at 180 °C for one minute, the temperature at which gelation began was noted.

#### 2.2.3. Rheological Analyses of Poly(PDMS/PEG/PPG Urethane) Based Thermogels

The rheological analyses of poly(PDMS/PEG/PPG urethane)-based thermogels were conducted on a Discovery DHP-3 hybrid rheometer (TA Instruments, New Castle, DE, USA) with a smooth steel concurrent geometry (width = 40 mm, interval = 0.9 mm) [30,32,33]. The mechanical properties of thermal gels during the process of solution-gel migration (rapid warming up in the range of 25–37 °C) were examined by scanning measurements of oscillation time at a fixed stretch (1%) as well as frequency (1 Hz). The mechanical characteristics of thermogels were also investigated by frequency sweep at human body temperature (37 °C). The oscillation variation of stroke varies between 0.01 and 100% at 1 Mhz, and the fixed oscillation strain is 1% at frequencies ranging between 0.01 and 100 Hz. Lastly, temperature ramp tests were conducted between 4 and 80 °C with a heating rate of 5 °C/min at the fixed strain (1%) and frequency (1 Hz) in order to study the changes in the mechanical characteristics of the thermal gels depending on the elevated temperature.

#### 2.2.4. Preparation of Poly(PDMS/PEG/PPG Urethane)-Based Thermogels Loaded with DOX

Firstly, phosphate buffered saline (PBS) wa used to dissolve DOX to a concentration of 2 mg/mL, then the block copolymer (DEP-*n*, 150 mg) was added into the DOX solution and mixed well. In details, the mixture was stirred at 40 rpm for 30 min at room temperature to dissolve completely, and equilibrated at 4 °C overnight, then formed into a gel at 37 °C (water bath). Eventually, the product of hydrogel (poly(PDMS/PEG/PPG urethane)/DOX) was fabricated at the concentration of DOX of 2 mg/mL and 15% concentrations of copolymers (*w*/*v*) and subjected to the following experiments [33].

#### 2.2.5. In Vitro Release Study from Poly(PDMS/PEG/PPG Urethane)-Based Thermogels Loaded with DOX

Poly(PDMS/PEG/PPG urethane)/DOX complex (containing 0.8 mg of DOX) was added to a 2 mL EP tube and pre-incubated for 5 min under 37 ± 5 °C to create an aqueous gel and equilibrate the tube in a water bath. Equal concentrations of free DOX solution were prepared simultaneously as a control. Two times the volume of pre-warmed PBS (pH = 6.5) (with 1% (*w*/*v*) of Tween 80) was added to the formed hydrogel, and releasing activity was conducted under 37 °C as well as at 50 rpm. The supernatant was collected for each time point, gently adding fresh PBS.

#### 2.2.6. Cell Culture and Preparation of Polymeric Micelles

HepG2 was acquired by American Type Culture Collection (ATCC). The cells of HepG2 were incubated in Dulbecco’s Modified Eagle Medium (DEME) supplemented with 100 U/mL of penicillin, 10% of fetal bovine serum (FBS) and 100 μg/mL of streptomycin sulfate in an incubator with 5% CO_2_ at 37 °C. DOX and block copolymer were dissolved in ethanol solution (weight ratio = 1:10); the clarified solution was added drop- wise to the PBS under ultrasonic conditions. The ethanol was removed with a nitrogen current. The following experiments were conducted.

#### 2.2.7. Cell Viability Assay

HepG2 cells were grown with a density of 5000 cells per well in 96-well plates. After incubation for 24 h with 5% CO_2_ at 37 °C, the cells were further cultured by treating them with freshly prepared poly(PDMS/PEG/PPG urethane)/DOX complex and single DOX solution at different concentrations in serum free medium for further culture. With 24 h treatment, a MTT solution (5 mg mL^−1^) was used to replace the medium and incubated for another period of 4 h with 37 °C. The supernatant was discarded and followed by the addition DMSO of 150 μL per well to dissolve the crystals. Finally, the absorbance at 492 nm ws measured to determine the viability of the cells.

#### 2.2.8. In Vitro Imaging of Cells/Confocal Imaging

HepG2 cells were cultured on 24-well plates at a density of 60,000 cells per well until cells were attached to the wall, then with free DOX solution and poly(PDMS/PEG/PPG urethane)/DOX complex in serum free medium for 1 h, 3 h, 9 h and 24 h in an incubator with 5% CO_2_ at 37 °C. The cells were treated with 4’, 6-diamidino-2-phenylindole (DAPI) staining for 5 min, and then washed three times with PBS to remove other impurities adsorbed on the cell surface. Then 4% paraformaldehyde solution fixation was used for 20 min before sealing the slides. The fluorescence photographs were taken and recorded with confocal laser scanning microscopy (CLSM).

#### 2.2.9. In Vivo Therapeutic Evaluation

The male BALB/c nude mice were housed in an individually ventilated cage (IVC) system and fed with sterilized food and water. Experiments on all animals were conducted in accordance with animal care guidelines and were authorized by the Animal Care and Use Committee of Xiamen University (XMULAC20190033). A model of HepG2 tumor in BALB/c nude mice was established. In detail, the HepG2 cells were transplanted into the subcutis of BALB/c nude mice at a density of 3 × 10^6^ cells per mouse. Once the tumor volume reached 200 mm^3^, the mice were treated with 100 μL of DOX or Poly(PDMS/PEG/PPG urethane)/DOX complex at its solution state by in situ injection. PBS and DEP-*n* only were injected for control groups. The volume of the tumor was calculated with vernier calipers and the formula (A × B^2^) × 0.5 was used, where A is the lengthiest diameter and with B being the minimum diameter. Seven days later, the mice were sacrificed and the tumors, heart, liver, spleen, lungs and kidneys of the mice were removed. 15% and 30% sucrose were used to dehydrate the tumors. Then the tumors and organ tissues were cut into frozen slides of 5 μm thickness and histologically studied with hematoxylin and eosin staining.

#### 2.2.10. Analysis of Statistics

All experimental data were expressed as mean ± standard deviation (SD). The data and charts as well as graphs were analyzed and processed by Origin 2018 (OriginLab, Northampton, MA, USA) and GraphPad Prism 8.0.2 (San Diego, CA, USA) analysis software for all experiments. Significance was analyzed using two-tailed Student’s t-test and one-way ANOVA. Statistically significant differences between the groups were represented by * for *p* < 0.05, ** for *p* < 0.01 and *** for *p* < 0.001, respectively.

## 3. Results

### 3.1. Poly(PDMS/PEG/PPG Urethane) Copolymers’ Synthesis and Characterisation

**Figure 1:**
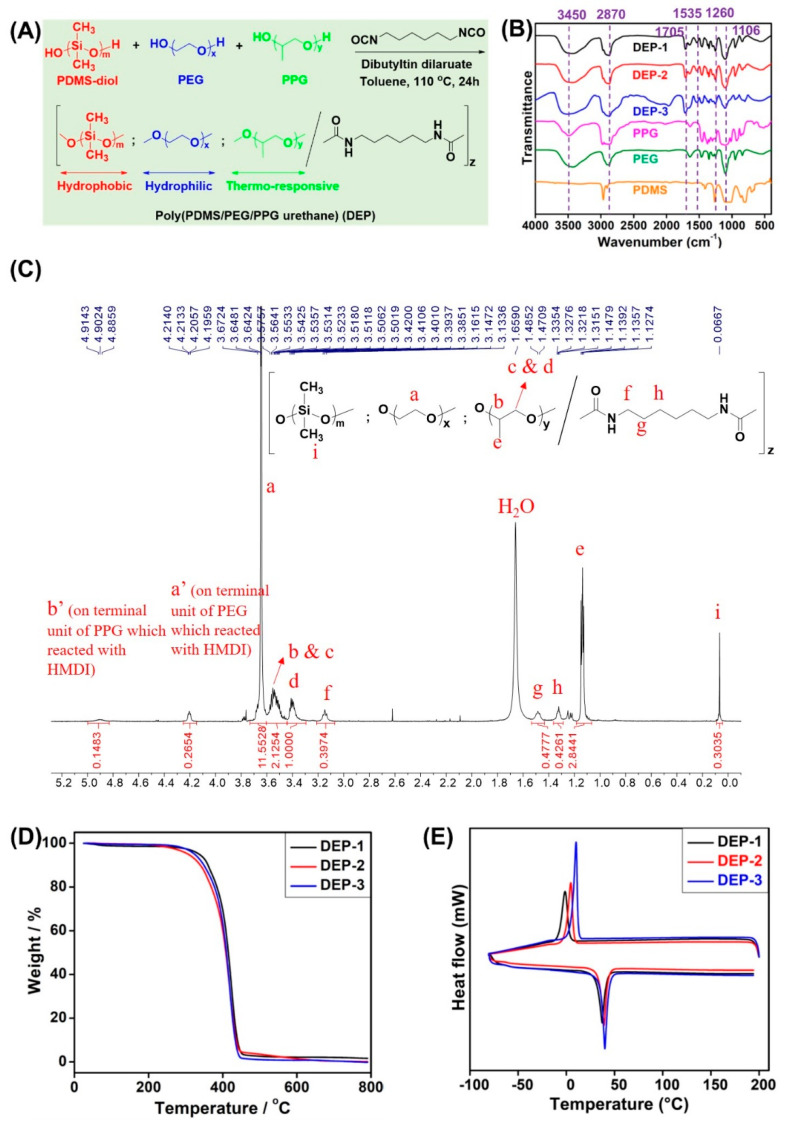
(**A**) The synthetic route of poly(PDMS/PEG/PPG urethane)s. (**B**) Normalized FT-IR spectra of starting materials PDMS-diol, PEG, PPG and synthesized copolymers DEP-*n*. (**C**) Typical ^1^H NMR spectrum of poly(PDMS/PEG/PPG urethane) (DEP-*2*) in room environment with CDCl_3_ as the solvent. (**D**) TGA and (**E**) DSC curves of DEP-*n* copolymers.

A novel amphiphilic copolymer poly(PDMS/PEG/PPG urethane) was meticulously designed and successfully synthesized in accordance with the synthetic route depicted in Figure 1A; with PEG and PPG as oligomeric polymers and PDMS ended with a diol group, a novel amphiphilic copolymer poly(PDMS/PEG/PPG urethane) was effectively created. In aqueous solution, the created copolymers can auto-form micelles that contain a hydrophobic PDMS core and a hydrophilic PEG shell. The PPG located in the inner hydrophobic core can have sol-gel change when affected by external temperature. It is intriguing to note that PDMS has certain antioxidant features and was deliberately designed to be located close to the PPG to provide a more stable hydrophobic core thereby facilitating the gel formation. Consequently, on account of the combination of antioxidant and temperature-sensitive functions, the obtained copolymer poly(PDMS/PEG/PPG urethane) can serve as a smart pharmaceutical transport carrier, which could compartmentalize the active drug in this form to be oxidized and improve the stability of the active drug.

The FT-IR analysis was also performed for all starting materials and the synthesized DEP-*n* copolymers. As shown in Figure 1B, the absorption peaks at 1705 cm^−1^ and 1535 cm^−1^ are characteristic peaks for the C=O and N-H bonds in the carbamate linkage, indicating a successful reaction between the hydroxyl terminated precursor and the isocyanate cross-linker. The presence of characteristic peaks of all starting materials in the final copolymers further proved the successful synthesis of the copolymers by comparing FT-IR spectra.

Figure 1C depicts the ^1^H NMR spectrum of poly(PDMS/PEG/PPG urethane), with the methylene proton of the PEG fragment being responsible for the signal at 3.64 ppm, the alkyne, methylene, and methyl protons of the PPG fragment being responsible for the signals at 3.6–3.4 ppm and 1.1 ppm, respectively, and the signal at 0.07 ppm was classified as a methylene proton of the PDMS-diol fragment. Simultaneously, the signals at 3.16–3.13, 1.48–1.47 and 1.32 ppm are methylene protons of the HMDI crosslinker, respectively, providing further evidence for polyurethane bond formation from the obtained copolymers.

The molecular weight (MW) and dispersion (ĐM) of the different percentages of poly(PDMS/PEG/PPG urethane) (DEP-*n* copolymer) were analyzed via gel permeation chromatography (GPC) and combined to the integral values of particular peaks from proton NMR spectra in order to calculate copolymer fragment compositions. It can be observed from Table 1 that the copolymers poly(2% PDMS/PEG/PPG polyurethane) (DEP-*1*) have the highest Mn~49.7 kDa, while poly(5% PDMS/PEG/PPG polyurethane) (DEP-*2*) and poly(8% PDMS/PEG/PPG polyurethane) (DEP-*3*) have Mn of 35.6 and 38.7 kDa, correspondingly. All copolymers had good ĐM~1.5, further confirming the satisfactory composition of poly(PDMS/PEG/PPG urethane).

The results of the TGA (Figure 1D) and DSC (Figure 1E) studies (Table 1) showed that an excellent thermal stability was exhibited by all the DEP-*n* copolymers, which degraded only above 300 °C (5% weight loss). Moreover, a single T_g_ value for each copolymer during heating was observed from the DSC curves, indicating a good miscibility of the polymers without microphase separation. In the meantime, an obvious thermal peak (T_m_) appears around 37–40 °C of heating due to the polymer block polymerization, caused by the melting of the PEG segment in the copolymer, which is lower than the Tm value of the pure PEG polymer.

### 3.2. Sol−Gel Transition Behaviors of DEP-n Copolymers

**Figure 2:**
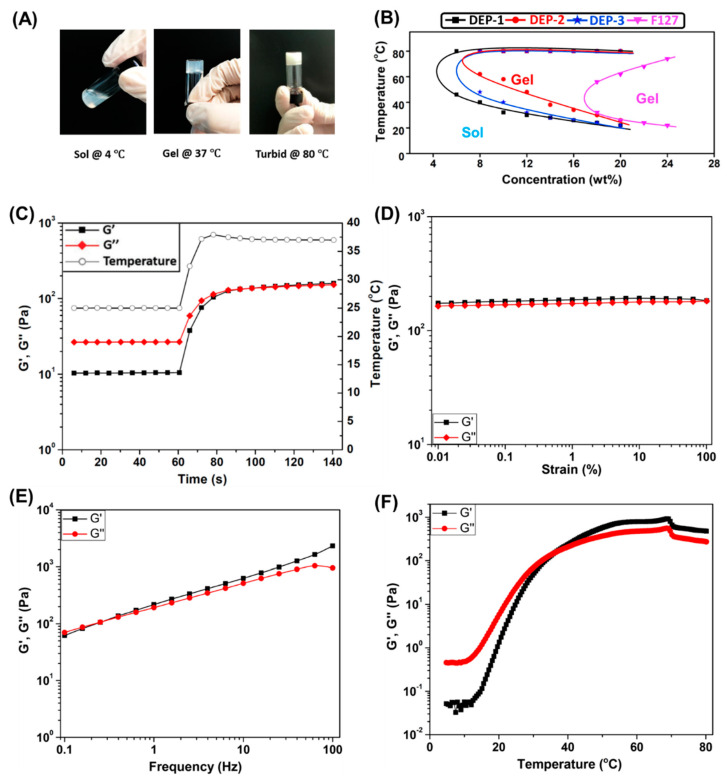
(**A**) Photo illustration of sol-gel transition of copolymer DEP-*2* at concentration of 15% (*w*/*v*); (**B**) Phase diagrams of copolymers DEP-*n* and F127. Rheological characteristics of DEP-2 on the basis of thermal gels (15% (*w*/*v*)). (**C**) Sweep diagram of oscillation time, i.e., oscillation time sweep at 25 °C for 60 s, then a rapid warming rate from 25 to 37 °C within 20 s and another 60-s oscillation time sweep at 37 °C to obtain the diagram. Oscillation (**D**) strain and (**E**) sweep diagram of frequency. (**F**) Ramp diagram of temperature.

In this report, the structural composition of the designed DEP-*n* copolymer contains hydrophobic PDMS, hydrophilic PEG and thermosensitive PPG components, of which PPG facilitates the formation of thermogel systems. The sol-gel transition behavior of the DEP-*n* copolymer thermogel between temperatures 4 and 80 °C was systematically measured by the inverted bottle method, and the phase diagram of the obtained copolymer was compared with that of copolymer F127. It can be seen from Figure 2B and Table 2 that the critical micelle concentration (CMC) of all DEP-*n* copolymers was essentially the same on the phase diagram and from the data studied, all were able to form gels above a certain concentration with critical gelation concentrations (CGC) of 6, 8 and 8% (*w*/*v*), respectively. All three copolymers have relatively similar gelation abilities and all are much better than the conventional F127 copolymer with a CGC of 18% (*w*/*v*). The DEP-*1* copolymer showed the best gelation performance compared to the DEP-*n* copolymer, followed by DEP-*3* and DEP-*2*. At the same concentration, the DEP-*1* copolymer had the lowest CGC and lower critical gel temperature (CGT), which may be caused by the concerted action of relatively high molecular weight, appropriate hydrophobic/hydrophilic component ratio together steric hindrance of the propyl side chain. Meanwhile, the thermal gels based on DEP-*n* copolymers exhibited a reversible sol-gel conversion feature, forming gels with sufficient strength at elevated temperatures, cloudy gel formed without break at up to 80 °C (Figure 2A).

### 3.3. Rheological Analyses of Poly(PDMS/PEG/PPG Urethane) Based Thermogels

It is theorized that the CGT of thermal gels decreases with increasing concentration, and CGT is adjusted to accommodate different application requirements. The present study is designed to form the gel under body temperature conditions at 37 °C in situ for drug delivery localization and sustained release, as well as to maintain the solvated state at room temperature to facilitate injection. Simultaneously, the gel should not be too robust to avoid incomplete drug release. Consequently, a DEP-*2* copolymer with a concentration of 15% (*w*/*v*) was selected as a carrier for the delivery of the chemotherapeutic drug DOX in this work, and the following studies were performed. Additionally, the rapid sol-gel process of 25 up to 37 °C showed by oscillation time scan that the modulus of storage G′ was slightly greater than the modulus of loss G″ at 37 °C, indicating that the DEP-*2* thermogel reacts rapidly, readily forms soft gels at body temperature, and is appropriate as in situ drug delivery system (Figure 2C). The DEP-*2* thermogel was furthermore analyzed by oscillatory strain and frequent scans to test the strength and stability of its increased shear stress, as well as frequency. As shown in Figure 2D, the viscoelasticity of the DEP-*2* thermogel at 37 °C is located in the linear range with a maximum strain of 100% and G′ of 180 plus Pa, which proves that the thermogel can maintain a stable gel state under an oscillatory stress of 260 Pa. According to the results of the oscillation frequency scan (Figure 2E), both G′ and G″ of the thermal gel increased significantly with increasing frequency, where the G′ value increased between 62 and 2330 Pa and the G″ value increased between 70 and 956 Pa in the test range of 0.1–100 Hz. The results indicate that the DEP-*2* thermal gel remains in a sol-gel state at frequencies below 0.4 Hz, it gradually forms a gel and becomes more viscous as the frequency increases. Therefore, the results of the above experiments further demonstrate that DEP-*2* thermal gels can maintain a gel state in a cellular dynamic microenvironment and be suitable for injectable drug delivery systems. Likewise, the results of Figure 2F show that G′ and G″ of DEP-*2* thermogels raise with increasing temperature, and the intersection of G′ and G″ values is the threshold sol-gel shift in the thermal gel. Thus, the critical sol-gel shift temperature for DEP-*2* thermal gels is 36.3 °C, and the G′ is always above the G″ value when the temperature is higher than this temperature, i.e., it is further demonstrated that the gel state of DEP-*2* thermogel can be maintained at 80 °C.

### 3.4. Study of In Vitro DOX Release of the Thermogel and HepG2 Cell Growth Inhibition

**Figure 3:**
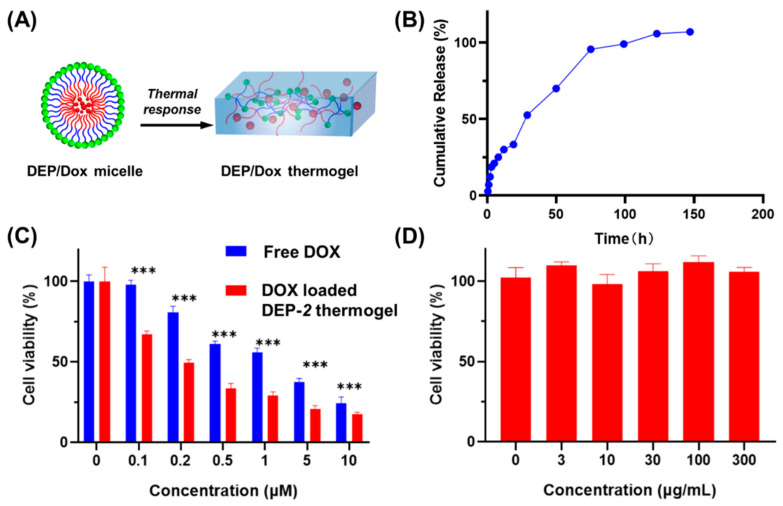
(**A**) Diagram of temperature-controlled conversion from polymer solution to hydrogel. (**B**) In vitro drug cumulative release of DOX from the DOX loaded DEP-*2* based thermogel at 37 °C. (**C**) Survival rate of cells processed using free DOX and DEP-*2* based thermal gels loaded with DOX. *** moreover, the thermal gels inhibited the proliferation of HepG2 cells remarkably more than free DOX. (**D**) Viability of cells treated by DEP-*2*.

In this report, a DEP-*2* thermogel was designed to encapsulate the classical antitumor drug DOX to obtain DEP-*2*@DOX copolymer solution, and DEP-*2* thermogel was formed by temperature increase (Figure 3A). With the short half-life and rapid elimination of DOX in vivo, it is necessary to increase the drug dose and multiple injections to obtain favorable efficacy in clinical tumors. However, multiple dosing leads to increased toxic side effects in other organs and enhanced damage to normal cells and tissues, thereby severely limiting the clinical application of DOX. In this work, we designed the thermal in situ gel to achieve sustained release and extend the half-life of DOX while ensuring injectability. Cumulative in vitro drug release assays were conducted to assess sustained release ability of DOX-loaded DEP-*2* thermal gel. The cumulative release profile of DEP-*2*@DOX thermal gel was performed in combination with the acidic microenvironment of the tumor, simulating slightly acidic conditions (pH 6.5). As shown in Figure 3B, the DEP-*2*@DOX thermosensitive gel was released at a 20% abrupt release rate within 5 h, followed by a continuous slow release, and reached complete release on day 6. These results revealed the effective encapsulation of DOX with DEP-*2* copolymers, while the incorporation of PDMS enhanced the structural stability of DEP-*2*@DOX micelles, resulting in a more durable sustained release of the drug.

To evaluate the antitumor activity and safety of DOX-containing polymeric micelles, various concentrations of both DOX solution and polymeric solutions of DEP-*2*@DOX were applied to HepG2 cells. As shown in Figure 3C, the antitumor activity of DEP-*2*@DOX group was obviously greater than that of the free DOX group. The result indicated that the DOX-containing DEP-*2*@DOX group showed enhanced antitumor activity, which may benefit from DOX localized and sustained release. The safety of DEP-*2* was confirmed by the cellular activity assay of HepG2 cells. As shown in Figure 3D, the safety of DEP-*2* copolymer vector was excellent in the concentration range of 0~300 μg mL^−1^.

### 3.5. In Vitro Cellular Uptake

**Figure 4:**
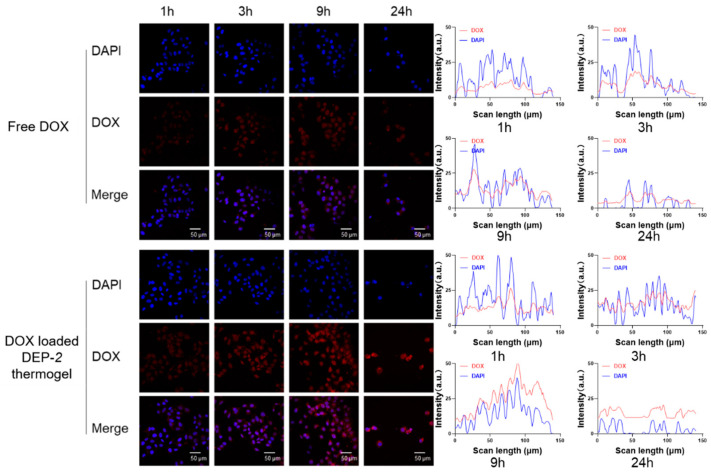
Uptake of various pharmaceutical preparations by HepG2 cells at 1, 3, 9 and 24 h.

Next, to verify that the DEP-*2* copolymer could serve as a good drug delivery platform, the drug delivery ability and cellular uptake of DEP-*2*@DOX copolymer micelles in HepG2 tumor cell lines were evaluated (Figure 4). DEP-*2*@DOX copolymer was more likely to enter HepG2 than free DOX. In detail, the DEP-*2*@DOX thermosensitive copolymer micelles group showed a favorable accumulation of red fluorescence signal of DOX after 3 h; it was indicated that the presence of DEP-*2* thermosensitive copolymer conferred intracellular drug delivery of DEP-*2*@DOX copolymer micelles. We speculate that PDMS-mediated drug retardation and PPG thermosensitive-mediated drug retention have a synergistic effect on this drug carrier. It should be noted that the fluorescence aggregation in the DEP-*2*@DOX group was significant after 9 h, which adequately confirmed the drug delivery ability and cellular uptake of the DEP-*2*-containing copolymer micelles. This result indicated that the thermo-sensitive polymeric micelles containing DEP-*2* could be taken up by HepG2, providing a possibility for the treatment of hepatocellular carcinoma.

### 3.6. In Vivo Therapy Study

**Figure 5:**
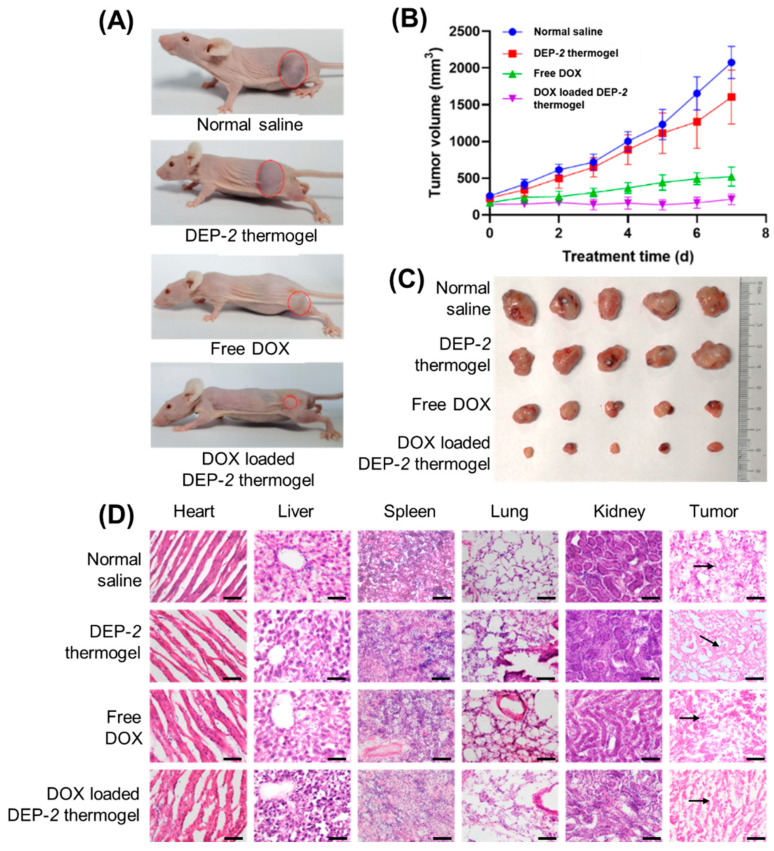
The in vivo therapeutic effect of DOX loaded DEP-*2* thermogel in nude mice liver cancer model. (**A**) Images of subcutaneous tumor dimensions in each therapy group (normal saline, DEP-*2* thermogel, free DOX and DOX loaded DEP-*2* thermogel treatment groups). (**B**) Tumor volume development curves (*n* = 5). (**C**) Tumor images of each group after excision from mice. (**D**) Histological examinations using HE staining (40×) was performed on anatomical tumors and organs such as heart, liver, spleen, lung and kidney on mice processed in normal saline, DEP-*2* thermogel, free DOX and DOX loaded DEP-*2* thermogel.

We further evaluated the efficacy of tumor treatment with DEP-*2*@DOX in a HepG2 tumor-carrying model (Figure 5A). Once the volume of the tumor was approximately 100 mm^3^, saline, DEP-*2* polymer, free DOX solution and DEP-*2*@DOX were administered intravenously to mice at the designed monitoring times. As shown in Figure 5B, DEP-*2*@DOX significantly inhibited tumor growth, with a reduction of approximately more than 60% compared to the saline or DEP-*2* polymer treated groups. At the end of the in vivo experiment, the size of the resected tumors was similar to the tumor volume measurement (Figure 5C). Hematoxylin and eosin (H&E) staining also verified the efficacy, with an increase in necrotic cells in the DEP-*2*@DOX group compared with saline or DEP-*2* polymer only (Figure 5D). In detail, the DEP-*2*@DOX group showed good anti-tumor effects with extensive necrosis of cancer cells (nuclei shown by arrows) shown by H&E staining under light microscopy. In addition, the H&E assay was used to assess the biological toxicity of these nanoparticles. There was a slight inflammation in the heart and liver tissues characterized by myocardial streaking and sinus tract disruption. However, mice treated with both saline and DEP-*2* showed no obvious signs of damage or toxicity. Meanwhile, the DEP-*2*@DOX group showed neither significant signs of injury nor toxicity in the pathological analysis of lung, spleen and kidney. In summary, these in vivo results suggest that DEP-*2* can be used as a drug delivery platform for thermosensitive in situ gels to enhance antitumor efficacy with minimal side effects.

## 4. Conclusions

In summary, this study reports a PDMS-PEG-PPG-based thermosensitive copolymer with sustained drug release by in situ formation of hydrogel, which can be used as a delivery vehicle for antitumor drugs. The efficient encapsulation of the clinical classical antitumor drug DOX provides an excellent strategy for the treatment of liver cancer. To the best of our knowledge, this is the first report of the therapeutic effect of supramolecular copolymer micelles employing PDMS-PEG-PPG delivery of DOX for hepatocellular cancer. The DEP-2@DOX thermosensitive copolymer micelles achieve good sustained drug release, enhance DOX’s therapeutic efficacy in hepatocellular carcinoma, and reduce DOX’s cardiotoxicity, according to both in vitro and in vivo research results. Furthermore, our work highlights the importance of PPG thermosensitizers in the formation of hydrogels in situ and the beneficial value of a sustained drug release strategy for clinical translation.

## Figures and Tables

**Table 1:** Characterization Summary of PDMS Incorporated Polyurethanes DEP-*n*.

Sample	Feed Ratio/g			Composition in Copolymer/wt% ^a^			Thermal Analysis				GPC Analysis	
	PDMS	PEG	PPG	PDMS	PEG	PPG	T*_m_*/°C ^c^	T*_g_*/°C ^c^	T*_c_*/°C ^c^	T*_d_*/°C ^b^	M*_n_*/Da	*Đ_M_*
DEP-*1*	0.2	6.53	3.27	0.5	74.6	24.9	37.2	−71.9	−1.3	336.7	49,669	1.47
DEP-*2*	0.5	6.33	3.17	1.3	73.4	25.3	39.1	−60.8	4.7	307.0	35,629	1.57
DEP-*3*	0.8	6.13	3.07	2.2	75.6	22.2	39.9	−61.8	10.1	324.2	38,654	1.57

^a^ Composition of purified copolymers calculated from integration of specific peaks in ^1^H NMR spectra; ^b^ T_d_ results from TGA analysis; ^c^ T_m_, T_c_ and T_g_ results with DSC analysis.

**Table 2:** Characterization summary of PDMS incorporated polyurethanes DEP-*n*.

Sample	CMC/g mL^−1^	CGC/(*w*/*v*)%	CGT at 12 (*w*/*v*)%/°C
DEP-*1*	3.379 × 10^−4^	6	30
DEP-*2*	3.564 × 10^−4^	8	48
DEP-*3*	3.448 × 10^−4^	8	32

## Data Availability

Not applicable.
